# Supplementary material for: Neural markers of suppression in impaired binocular vision
Source: Neuroimage. 2021 Apr 15;230:117780. doi: 10.1016/j.neuroimage.2021.117780 (PMC8063178; doi:10.1016/j.neuroimage.2021.117780)
Supplement: Supplementary file 1 [file mmc1.pdf]

Supplementary materials

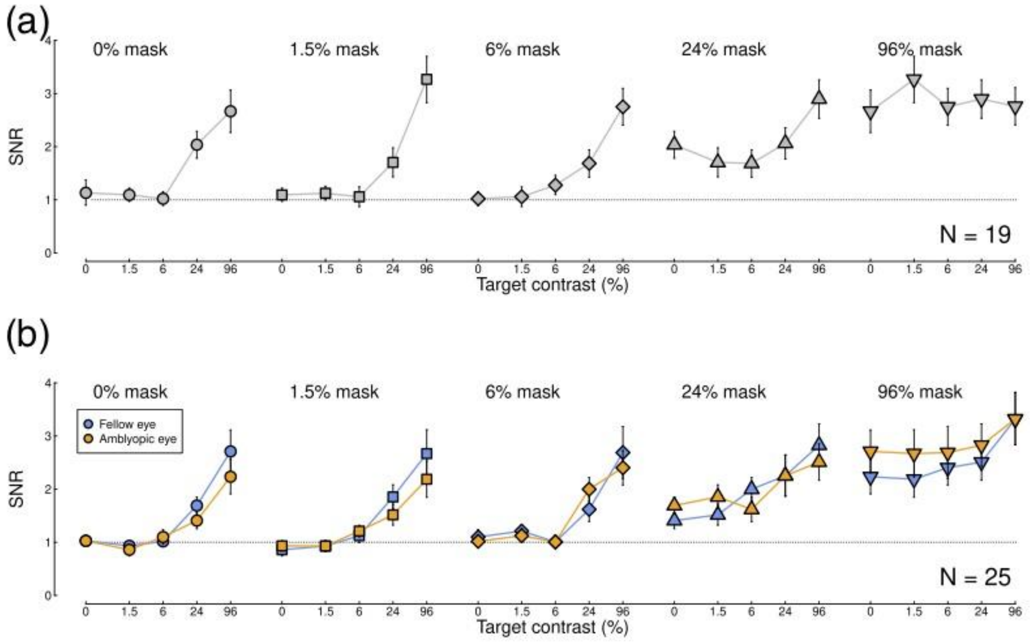

Figure S1: SSVEP data for the second harmonic (2F) response. The main trends are the same as in Figure 5c,d, though the SNRs are overall lower (note y-axis scaling). Panel (a) shows data from control participants (N=19), and panel (b) shows data from patients (N=25).

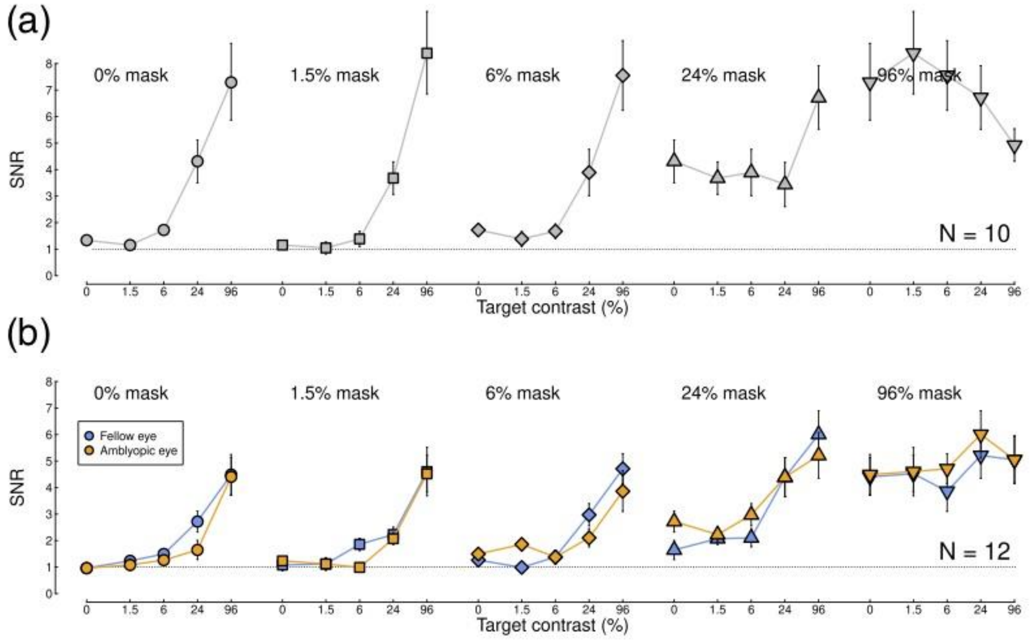

Figure S2: SSVEP data for only the participants who completed the fMRI experiment. The main trends are the same as in Figure 5c,d. Panel (a) shows data from control participants (N=10), and panel (b) shows data from patients (N=12). For the control participants, ANOVA revealed main effects of left eye contrast (F(4,36) = 22.27, $p < 0.001$, $\omega^2 = 0.36$), right eye contrast (F(4,36) = 16.87, $p < 0.001$, $\omega^2 = 0.34$) and their interaction (F(16,144) = 5.67, $p < 0.001$, $\omega^2 = 0.24$). For the patients, ANOVA revealed main effects of fellow eye contrast (F(4,44) = 22.31, $p < 0.001$, $\omega^2 = 0.39$), amblyopic eye contrast (F(4,44) = 14.99, $p < 0.001$, $\omega^2 = 0.29$) and their interaction (F(16,176) = 3.22, $p < 0.001$, $\omega^2 = 0.10$). Pairwise comparisons between the fellow and amblyopic eyes in the 0% mask condition at 24% and 96% target contrasts were not significant (24%: $t = 1.78$, $df = 11$, $p = 0.10$, Cohen's $d = 0.51$; 96%: $t = 9.12$, $df = 11$, $p = 0.91$, Cohen's $d = 0.08$).
